# Large Artery Remodeling and Dynamics following Simulated Microgravity by Prolonged Head-Down Tilt Bed Rest in Humans

**DOI:** 10.1155/2015/342565

**Published:** 2015-01-13

**Authors:** Carlo Palombo, Carmela Morizzo, Martino Baluci, Daniela Lucini, Stefano Ricci, Gianni Biolo, Piero Tortoli, Michaela Kozakova

**Affiliations:** ^1^Department of Surgical, Medical, Molecular and Critical Area Pathology, University of Pisa, 56124 Pisa, Italy; ^2^Department of Medical Biotechnologies and Translational Medicine, University of Milan, 20129 Milan, Italy; ^3^Department of Information Engineering, University of Florence, 50139 Florence, Italy; ^4^Department of Medicine, Surgery and Health Sciences, University of Trieste, 34127 Trieste, Italy

## Abstract

The effects of simulated microgravity on the static and dynamic properties of large arteries are still mostly unknown. The present study evaluated, using an integrated vascular approach, changes in structure and function of the common carotid and femoral arteries (CCA and CFA) after prolonged head-down tilt bed rest (HDTBR). Ten healthy men were enrolled in a 5-week HDTBR study endorsed by the Italian Space Agency (ASI). Arterial geometry, flow, stiffness, and shear rate were evaluated by ultrasound. Local carotid pulse pressure and wave reflection were studied by applanation tonometry. After five weeks of HDTBR, CFA showed a decrease in lumen diameter without significant changes in wall thickness (IMT), resulting in an inward remodeling. Local carotid pulse pressure decreased and carotid-to-brachial pressure amplification increased. The ratio of systolic-to-diastolic volumetric flow in CFA decreased, whereas in CCA it tended to increase. Indices of arterial stiffness and shear rate did not change during HDTBR, either in CCA or CFA. In summary, prolonged HDTBR has a different impact on CCA and CFA structure and flow, probably depending on the characteristics of the vascular bed perfused.

## 1. Introduction

Prolonged head-down tilt bed rest (HDTBR) represents an established experimental model allowing investigating the physiologic adaptations to microgravity conditions on the ground [1]. Studies evaluating the effect of simulated microgravity on cardiovascular system have demonstrated that the prolonged HDTBR is followed by a significant decrease in left ventricular (LV) mass accompanied by a reduction in LV performance [2]. Our group has previously demonstrated that a reduction in echocardiographic indices of LV systolic and diastolic performance after a 5-week period of HDTBR does not reflect an impairment in intrinsic myocardial function, but simply an adaptive response to circulatory unloading [3]. Data regarding response of the arterial system to bed rest are less clear. Prolonged unloading has been shown to induce an inward remodeling of the femoral artery with time-dependent decrease in arterial size, reaching 17% after 52 days of bed rest [4]. Eight weeks of physical inactivity have been also shown to increase carotid and femoral artery wall thickness and wall-to-lumen ratio [5]. Pathophysiologic mechanisms underlying these structural changes are supposed to include inactivity-related muscle atrophy associated with a reduced metabolic demand of the downstream muscle tissue [6], as well as an impact of altered hemodynamic stimuli on the arterial wall. It has been demonstrated that arteries are capable to respond to changes in hemodynamic stimuli (flow and shear rate) and mechanical forces (circumferential and pulsatile stress) by modification of their geometry [7]. However, previous studies did not provide definite evidence on bed rest induced changes in flow, shear rate, or wall stress, and data regarding impact of deconditioning on arterial stiffness and wave reflection are sporadic [8]. In the present study, the common carotid and femoral arteries were investigated at baseline and after a 5-week HDTBR by an integrated vascular approach allowing evaluating impact of deconditioning on different structural and functional properties of the arterial system.

## 2. Methods

### 2.1. Subjects

Ten healthy young volunteers, all men, mean age 23 ± 2 years, were enrolled in a multidisciplinary HDTBR study endorsed by the Italian Space Agency (ASI) and taking place at the Orthopedic Hospital Valdoltra, Ankaran, Slovenia. None of the volunteers was a smoker. Medical history, physical examination, laboratory examinations, resting and stress ECG, and echocardiography have excluded any acute or chronic medical problem. The National Committee for Medical Ethics of the Slovene Ministry of Health (Ljubljana, Slovenia) approved the study. All participants were informed about the aim of the investigation, the procedures, and the methods and signed a written informed consent form according to the Declaration of Helsinki.

### 2.2. Study Protocol

All participants underwent a 5-week period of bed rest in a 6° head-down tilt position (HDTBR). During the bed rest period, participants were kept strictly in bed for 24 hours a day, and none of them took any medication or underwent any physical or pharmacological countermeasure. Dietary intake was 2300 kcal/day, and water intake was 1.0–1.5 L/day. Diuresis was monitored daily, and BP and heart rate were measured every 4 hours during daytime. Body composition and hematocrit were measured before and at the end of the bed rest. Carotid and femoral ultrasound, carotid applanation tonometry, carotid-femoral pulse wave velocity (PWV), and cardiac ultrasound were performed the day before entering bed rest and within 24 hours after its termination. Vascular and cardiac examinations were performed in a quiet room, three hours after a light breakfast and after an acclimatization period of 30 min in supine position. All vascular acquisitions and readings were performed by a single operator (CM).

### 2.3. Measurements

#### 2.3.1. Body Composition Assessment

Body weight and fat-free mass were measured by electrical bioimpedance (BioScan 916S; Maltron International Ltd., Essex, UK).

#### 2.3.2. Carotid and Femoral Ultrasound

On the right common carotid and femoral artery (CCA, CFA), two sequential acquisitions were performed using a modified commercially available equipment (MyLab30, Esaote, Firenze, Italy, with a 7.5–12 MHz broadband linear transducer, LA435), in order to obtain the following measures: (a) intima-media thickness (IMT), systolic, diastolic, and mean arterial luminal diameters; (b) centerline blood flow velocity (by conventional Duplex ultrasound); (c) shear rate values directly measured at the near and far arterial wall (by multigate Doppler system). For all ultrasound acquisitions, the angle of inclination for Doppler velocity measurements was consistently adjusted to 60°, whereas the vessel lumen was set parallel to the transducer.

(a) Longitudinal B-mode images of the right CCA and CFA with well-defined intima-media complex of the near and far wall were obtained and a loop over 5 cardiac cycles was stored. Brachial pressure and heart rate were measured during loop acquisition (Omron 705, Tokyo, Japan). Vascular ultrasound scans were analyzed by the computer-driven image analysis system MIP (Medical Image Processing; Institute of Clinical Physiology, CNR, Pisa, Italy); end-diastolic and end-systolic frames of the CCA or CFA were selected; end-diastolic far-wall IMT and minimum and maximum luminal diameters were measured within a region of interest. Arterial remodeling was assessed as a ratio of end-diastolic IMT and luminal radius (IMT/radius), where radius was calculated as minimum diameter/2. End-diastolic wall stress (kPa) was calculated as follows: diastolic BP (in kPa) ∗ end-diastolic radius/IMT. Delta diameter (Δ diameter) was calculated as the difference between maximum and minimum diameter, and the stiffness index beta was calculated as minimum diameter ∗ ln(Systolic BP/Diastolic BP)/Δ diameter. The values reported represent the average of three cardiac cycles. Intraindividual variability of IMT and arterial diameter measurement by MIP in our laboratory is 4.8 ± 2.8% and 3.1 ± 1.9%, respectively. To estimate mean volumetric flow per beat, CCA and CFA diameter averaged over the entire cardiac cycle was measured from the radiofrequency signal processed by a dedicated software tool (QIMT, Esaote Europe, Maastricht, Netherlands) in a 1 region of interest placed at the same area as flow-velocity integral was measured.

(b) In spectral Doppler recordings, peak systolic and diastolic velocities as well as systolic, diastolic and systo-diastolic flow-velocity integrals were measured, both for CCA and CFA. Resistive index was calculated as follows: (peak systolic velocity − peak diastolic velocity)/peak systolic velocity. Systolic and diastolic volumetric flows per beat were calculated as systolic and diastolic arterial area (Π∗diameter^2^/4) multiplied by the corresponding flow-velocity integral. Mean volumetric flow over cardiac cycle was calculated as systo-diastolic flow-velocity integral multiplied by area of luminal diameter averaged over cardiac cycle as obtained from radio-frequency signal (see above). All values are reported as the average of 3 cardiac cycles.

(c) Shear rate was assessed by a validated multigate Doppler system determining a flow velocity profile from a matrix of 128-point power spectral densities corresponding to 128 different depths along the Doppler beam [9]. A custom PC board based on a high-speed digital signal processor was used to process the quadrature demodulated echo signals derived from the MyLab30 and to display results in real time. A polynomial least-square fit is applied off-line on the 128 experimental velocity points, and the resulting profile is used to evaluate the gradient with respect to radius. The local peak shear rate at the near and far blood-wall interfaces was calculated.

#### 2.3.3. Carotid Applanation Tonometry

Carotid applanation tonometry was performed on the right CCA using a validated system (PulsePen; Diatecne, Milan, Italy) [10]. Carotid pressure waveforms were calibrated according to brachial mean and diastolic pressure as previously described [11]. In the carotid pressure waveform, the following parameters were measured: local systolic BP, local pulse pressure, and augmentation index (AIx). Pulse pressure index was calculated as local pulse pressure divided by mean BP and pulse pressure amplification as the ratio of brachial to carotid pulse pressure [12]. The mean of 3 measurements was used for statistical analysis.

#### 2.3.4. Carotid-Femoral Pulse Wave Velocity

Carotid-femoral PWV was measured according to current guidelines [13] using the Complior device (Alam Medical, Vincennes, France). Briefly, arterial waveforms were obtained transcutaneously over the right CCA and femoral artery, and the time delay (*t*) was measured between the feet of the two waveforms. The distance (*D*) covered by the waves was established as the distance between the two recording sites. PWV was then calculated as* D* (meters)/*t* (seconds). The measurement was performed three times and the mean value was used for statistical analysis. Simultaneous BP measurement was performed at the left brachial artery (Omron, Kyoto, Japan). In our laboratory, intraindividual variability of PWV measurement is 4.5 ± 2.8%.

#### 2.3.5. Cardiac Ultrasound

Cardiac ultrasound was performed as previously described [3]. Stroke volume was measured as a product of aortic area and flow-velocity integral in aortic orifice [14]. Flow-velocity integral was obtained also in ascending aorta from the suprasternal notch. Results on changes in LV mass, performance, and loading conditions observed in the same study group were previously published in detail [3].

### 2.4. Statistical Analysis

Quantitative data are expressed as mean ± sd. Paired *t*-test was used to compare the measurements obtained before and after HDTBR. Linear univariate regression analysis was used to test the relationships between bed rest-induced changes in arterial diameter or flow and in FFM or stroke volume. Statistical significance was set at a value of *P* less than 0.05. Statistical analysis was performed by JMP software, version 8.0.2 (SAS Institute Inc., Cary, North Carolina, USA).

## 3. Results

During the bed rest period, body weight, BMI, fat FFM and Doppler-derived stroke volume, and flow-velocity integral in ascending aorta diminished, peripheral BP did not change significantly, and heart rate and hematocrit increased (Table 1).

After 5 weeks of HDTBR, no significant changes were observed in CCA geometry and stiffness (Table 2). CFA diameter significantly decreased (minimum diameter by 10 ± 4%), CFA intima-media thickness did not change, and therefore, the ratio end-diastolic CFA IMT/radius increased and circumferential wall stress decreased (Table 2). The changes in CFA minimum diameter showed a trend to correlate with changes in fat-free mass (*r* = 0.49; *P* = 0.15). CFA beta stiffness index remained unchanged after HDTBR (Table 2).

Responses in flow velocities and volumes differed between CCA and CFA. In CCA, peak systolic and diastolic velocity did not change significantly during the bed rest period. In CFA, both peak systolic and diastolic velocities increased, but the increase was higher for diastolic velocity and, consequently, the resistive index decreased. Systolic volumetric flow per beat remained stable both in CCA and in CFA. In contrast, diastolic volumetric flow showed a trend to decrease in CCA, whereas it increased in CFA (Table 2). Consequently, the ratio of systolic-to-diastolic flow in CCA tended to increase, while in CFA it significantly decreased. The relationships between volumetric flow per beat in CCA and stroke volume or ascending aorta flow-velocity integral (estimated by Doppler echocardiography) as well as the relationship between volumetric flow per beat in CFA and stroke volume were tested. In CCA, the mean and diastolic flow per beat at baseline were strongly related to baseline stroke volume (*r* = 0.75; *P* = 0.01 and *r* = 0.82; *P* < 0.001), and the changes in mean and diastolic flow per beat during HDTBR were related to changes in stroke volume (*r* = 0.70; *P* < 0.05 and *r* = 0.54; *P* = 0.10), as well as to changes in ascending aorta flow-velocity integral (*r* = 0.78; *P* < 0.01 and *r* = 0.48; *P* = 0.15). None of these relationships were observed for CFA.

Wall shear rate at near and far arterial wall did not change during HDTBR either in CCA or in CFA (Table 2). In CCA, the mean luminal diameter was positively related to wall shear rate at anterior (*r* = 0.62; *P* = 0.05) and posterior wall (*r* = 0.63; *P* = 0.05); however, this correlation was lost after the period of bed rest. No relationship between shear rate and luminal diameter was observed for CFA.

Carotid femoral PWV and AIx did not change after 5 weeks of HDTBR, while local carotid pulse pressure and pulse pressure index decreased and pressure amplification index increased (Table 3). Changes in hematocrit were not related to changes in vascular measures.

## 4. Discussion

**Figure 1:**
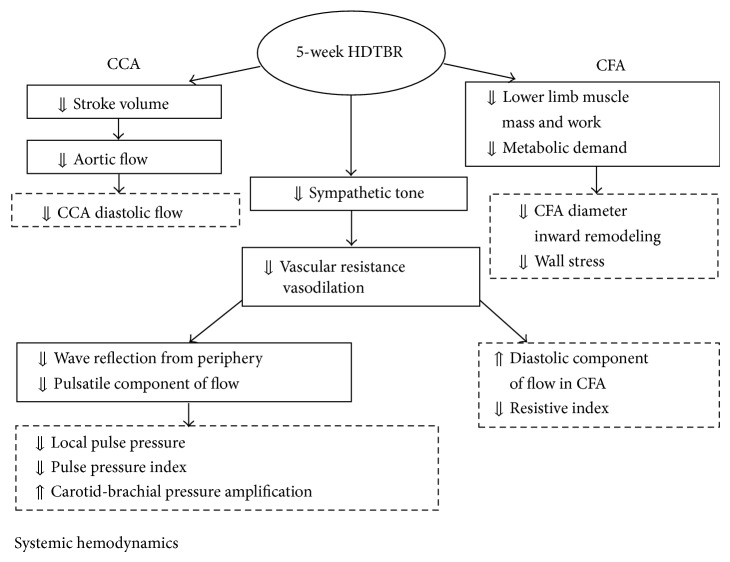
Schematic representation of changes observed at common carotid level, at femoral artery level, and in central hemodynamics after 35-days head-down tilt bed rest in 10 young healthy volunteers.

The present study compares the response of large elastic and muscular artery to prolonged HDTBR and provides some novel information about arterial mechanics and flow dynamics during deconditioning that are summarized in Figure 1. A complex vascular approach integrating established investigative modalities with new advanced techniques was exploited to this purpose.

### 4.1. Bed Rest Deconditioning and Vascular Geometry

In our young healthy volunteers, an inward remodeling of femoral artery, due to luminal diameter reduction, and a diminution of circumferential wall stress was observed after a 35-day bed rest. Carotid geometry, on the other hand, was not significantly influenced by deconditioning, a finding confirming the differences in response of carotid and femoral artery to bed rest. Observed reduction in femoral artery diameter is in agreement with results of the Berlin Bed Rest (BBR) study [4] and may reflect structural and/or functional changes, extensively discussed in a review paper of Thijssen et al. [7]. In our study, the changes in CFA diameter showed a trend to correlate directly with changes in fat-free mass. Such a correlation might suggest that the reduction in CFA lumen reflects a reduced metabolic demand in a downstream muscle tissue, as the gravitational unloading involves both artery and muscle. Yet, similar to the BBR study, femoral artery volumetric flow did not decrease after bed rest. This apparent discrepancy could be explained by the fact that conduit arteries adapt primarily to peak blood flow and oxygen demand during exercise [15]. The association between conduit artery diameter and muscle work has been suggested also in a recent study, in which a reduction in femoral artery diameter was demonstrated in subjects wearing a mechanical device (HEPHAISTOS) allowing an “unloaded orthosis” [16], that is, a reduction of muscle work with unchanged gravitational acceleration.

In contrast with results of the second BBR study [5] reporting an increment in CCA and femoral artery IMT after a 60-days bed rest period, we did not observe a significant change in carotid or femoral wall thickness; a shorter duration of bed rest in our study could explain the discrepancy.

### 4.2. Bed Rest Deconditioning and Blood Flow

Previous studies evaluating the effect of unloading on the blood flow in the lower extremity have produced inconclusive evidence. In the BBR study [4], the mean blood flow did not change in CFA and superficial femoral artery after bed rest; in the HEPHAISTOS study [16], blood flow volume in superficial femoral artery remained unaffected by a reduction in muscle work while flow velocity increased by 17%; in studies using plethysmography, the blood flow at the arteriolar level decreased [17]. In a HDTBR study, a large portion of blood flow reduction as measured by plethysmography was observed already after the first day of unloading [18]. However, plethysmographic and Doppler measurements are hardly comparable.

Our study is the first to look separately at the systolic and diastolic flow velocities and volumes, both at CCA and CFA levels. In CCA, the diastolic component of volumetric flow after HDTBR showed trend to decrease, and the changes in both diastolic and mean volumetric flow were directly related to changes in stroke volume and in flow-velocity integral in ascending aorta. This observation suggests that carotid artery flow simply mirrors the changes occurring in aortic flow. In contrast, in CFA, the diastolic component of local blood flow significantly increased and, consequently, the resistive index and the ratio of systolic to diastolic flow volume decreased. This behavior may reflect a decrease in local vascular resistance at arteriolar level of the leg. Based upon evidence from previous HDTBR studies, a reduction of sympathetic firing to lower limb vessels could explain our finding. Stout et al. reported that during simulated microgravity, a cutaneous microcirculatory vasodilation is more marked in the lower than in the upper part of the body, being related to a baroreflex-mediated withdrawal of a sympathetic tone [19]. More recently, in healthy volunteers maintained for 90 days in HDTBR, Ferretti et al. demonstrated a significant reduction of the efferent muscle sympathetic nerve activity in the leg [20].

Wall shear rate, describing the tangential force exerted by the flow stream on the arterial wall, did not change during the study, either in elastic or muscular artery. A role of shear rate in arterial diameter control was suggested by a direct relationship between near- and far-wall shear rate and CCA luminal diameter at baseline conditions [21]. However such a relationship was not observed at femoral artery level.

### 4.3. Bed Rest Deconditioning, Large Artery Stiffness, and Central Pressure

The lack of bed rest induced changes in indices of either local carotid and femoral stiffness or segmental aortic stiffness (Table 3) further supports the premise that the changes in large artery geometry and flow depend upon functional instead of structural vascular changes. The significant reduction in the local carotid pulse pressure, a good surrogate of aortic pressure [22], together with the reduction in the pulse pressure index and the increase in the carotid-to-brachial pressure amplification estimated by means of carotid waveform analysis, reflect a significant decrease in the pulsatile component of central pressure compared to the steady one and, possibly, a reduction of wave reflection from a vasodilated periphery.

## 5. Study Limitations

This study has several limitations. First, the population studied is small and consists only of men. Second, all participants were young, and thus the results do not provide information on the effect of bed rest on arterial structure and function in older subjects. Third, vascular examinations were performed only one day before and one day after HDTBR; consequently, we could not evaluate a sequence of changes over the bed rest period or after termination of bed rest. Fourth, plasma viscosity was not measured, so that only wall shear rate but not wall shear stress could be assessed. Furthermore, the experimental model used in this study, although established for simulating unloading conditions related to microgravity, does not allow separating the effects of a reduced muscle activity from those of a reduced gravitational acceleration. Finally, AIx values were not adjusted for heart rate. Due to the significant increase in heart rate observed after bed rest, we could have overestimated AIx and underestimated a reduction in wave reflection.

## 6. Conclusion

An integrated vascular approach combining established and experimental ultrasound, arterial contour wave analysis, and pulse wave velocity assessment was exploited to investigate the adaptation of large arteries to microgravity conditions simulated by HDTBR. Prolonged HDTBR showed a different impact on CCA and CFA structure and flow, probably depending on the characteristics of the vascular bed perfused. Changes in CCA blood flow seem to reflect bed rest induced decrease in stroke volume and aortic flow and did not alter CCA geometry. Reduction in CFA luminal diameter and inward remodeling may result from reduced metabolic demand in a downstream unloaded muscle tissue, and changes in CFA flow may reflect decrease in local vascular resistance secondary to withdrawal of a sympathetic tone. Observed changes in systemic hemodynamics that included decrease in local pulse pressure and pulse pressure index and increase in carotid-brachial pressure amplification suggest a reduction of wave reflection from a vasodilated periphery (Figure 1). Therefore, 5-week HDTBR results in a relative reduction of the pulsatile versus the steady component of blood flow and arterial pressure, possibly reflecting changes in systemic hemodynamics and in sympathetic control of the arteriolar tone.

In the prospect of improving the management of subjects undergoing real microgravity conditions, data obtained in this study confirm the indication to active counter-measurements aimed to prevent unloading-related sarcopenia as well as the possible usefulness of common carotid artery as a “window” to monitor central hemodynamic changes.

## Figures and Tables

**Table 1:** Main anthropometric and hemodynamic characteristics and hematocrit in 10 healthy volunteers before and after HDTBR.

	Before	After	*P*
Weight (kg)	75 ± 10	73 ± 9	*<0.01 *
BMI (kg/m^2^)	23.3 ± 2.0	22.8 ± 1.6	*<0.05 *
Fat-free mass (kg)	64 ± 5	61 ± 5	*<0.0001 *
Hematocrit (%)	44.4 ± 2.9	47.9 ± 2.1	*0.001 *
Systolic BP (mmHg)	115 ± 17	113 ± 10	*0.51 *
Diastolic BP (mmHg)	62 ± 7	65 ± 4	*0.33 *
Pulse pressure (mmHg)	53 ± 11	48 ± 10	*0.19 *
Heart rate (bpm)	60 ± 10	71 ± 7	*<0.005 *
Stroke volume (mL)	76 ± 11	63 ± 10	*<0.01 *
FVI ascending aorta (cm)	21.7 ± 2.1	19.2 ± 2.8	*0.01 *

BMI: body mass index; FFM: fat-free mass; BP: blood pressure; FVI: flow-velocity integral.

**Table 2:** Common carotid artery and common femoral artery structure, stiffness, and flow before and after HDTBR in 10 healthy volunteers.

	CCA		*P*	CFA		*P*
	Before	After		Before	After	
IMT (*μ*m)	503 ± 48	520 ± 36	*0.27 *	515 ± 79	523 ± 57	*0.58 *
Diameter minimum (mm)	5.1 ± 0.3	5.0 ± 0.3	*0.12 *	7.4 ± 0.9	6.7 ± 1.0	*<0.01 *
Diameter maximum (mm)	5.9 ± 0.3	5.8 ± 0.3	*0.09 *	8.1 ± 1.0	7.4 ± 1.0	*<0.01 *
Δ diameter (mm)	0.80 ± 0.13	0.78 ± 0.11	*0.71 *	0.74 ± 0.22	0.72 ± 0.23	*0.72 *
End-diastolic IMT/radius	0.18 ± 0.02	0.19 ± 0.02	*0.14 *	0.14 ± 0.03	0.16 ± 0.02	*<0.01 *
End-diastolic wall stress (kPa)	42.4 ± 5.9	41.5 ± 5.6	*0.70 *	60.7 ± 10.9	54.9 ± 7.9	*0.05 *
Beta index	3.2 ± 0.7	2.9 ± 0.7	*0.29 *	6.5 ± 1.9	5.6 ± 2.1	*0.20 *
Peak velocity systolic (cm/s)	124 ± 25	125 ± 22	*0.83 *	89 ± 15	116 ± 35	*0.09 *
Peak velocity diastolic (cm/s)	25 ± 5	26 ± 6	*0.66 *	6 ± 3	10 ± 5	*<0.05 *
Resistive index	0.79 ± 0.04	0.79 ± 0.04	*0.88 *	0.94 ± 0.02	0.91 ± 0.02	*0.05 *
Mean flow per beat (mL)	9.3 ± 1.6	8.6 ± 1.3	*0.42 *	9.4 ± 2.1	9.3 ± 2.4	*0.90 *
Systolic flow per beat (mL)	4.9 ± 0.9	5.0 ± 0.6	*0.74 *	7.8 ± 2.1	7.1 ± 2.3	*0.37 *
Diastolic flow per beat (mL)	3.4 ± 0.7	2.9 ± 0.6	*0.08 *	1.5 ± 0.5	1.9 ± 0.7	*0.05 *
Ratio syst/diast flow per beat	1.5 ± 0.5	1.7 ± 0.4	*0.07 *	5.8 ± 2.0	3.9 ± 0.6	*0.01 *
WSR peak near wall (s^−1^)	524 ± 80	575 ± 120	*0.29 *	569 ± 177	557 ± 197	*0.89 *
WSR peak far wall (s^−1^)	460 ± 107	494 ± 95	*0.49 *	357 ± 52	326 ± 69	*0.34 *

IMT: intima-media thickness; WSR: wall shear rate.

**Table 3:** Carotid-femoral pulse wave velocity and carotid pressure waveform analysis before and after HDTBR in 10 healthy volunteers.

	Before	After	*P*
C-F PWV (m/s)	6.9 ± 1.0	6.9 ± 0.7	*0.53 *
Local SBP (mmHg)	106 ± 11	101 ± 7	*0.23 *
Local PP (mmHg)	44 ± 11	36 ± 7	*<0.05 *
PPI	0.55 ± 0.11	0.46 ± 0.09	*0.05 *
AIx	6.6 ± 5.9	5.4 ± 4.4	*0.50 *
Pressure amplification	1.24 ± 0.11	1.31 ± 0.10	*<0.05 *

C-F PWV: carotid-femoral pulse wave velocity; SBP: systolic blood pressure; PP: pulse pressure; PPI: pulse pressure index; AIx: augmentation index.

## References

[1] Fortrat J.-O., Sigaudo D., Hughson R. L., Maillet A., Yamamoto Y., Gharib C. (2001). Effect of prolonged head-down bed rest on complex cardiovascular dynamics. *Autonomic Neuroscience: Basic and Clinical*.

[2] Perhonen M. A., Zuckerman J. H., Levine B. D. (2001). Deterioration of left ventricular chamber performance after bed rest: “cardiovascular deconditioning” or hypovolemia?. *Circulation*.

[3] Kozàkovà M., Malshi E., Morizzo C. (2011). Impact of prolonged cardiac unloading on left ventricular mass and longitudinal myocardial performance: an experimental bed rest study in humans. *Journal of Hypertension*.

[4] Bleeker M. W. P., de Groot P. C. E., Rongen G. A. (2005). Vascular adaptation to deconditioning and the effect of an exercise countermeasure: results of the Berlin Bed Rest study. *Journal of Applied Physiology*.

[5] van Duijnhoven N. T. L., Green D. J., Felsenberg D., Belavý D. L., Hopman M. T. E., Thijssen D. H. J. (2010). Impact of bed rest on conduit artery remodeling: effect of exercise countermeasures. *Hypertension*.

[6] de Groot P. C. E., Bleeker M. W. P., Hopman M. T. E. (2006). Magnitude and time course of arterial vascular adaptations to inactivity in humans. *Exercise and Sport Sciences Reviews*.

[7] Thijssen D. H. J., Green D. J., Hopman M. T. E. (2011). Blood vessel remodeling and physical inactivity in humans. *Journal of Applied Physiology*.

[8] Nosova E. V., Yen P., Chong K. C. (2014). Short-term physical inactivity impairs vascular function. *Journal of Surgical Research*.

[9] Tortoli P., Morganti T., Bambi G., Palombo C., Ramnarine K. V. (2006). Noninvasive simultaneous assessment of wall shear rate and wall distension in carotid arteries. *Ultrasound in Medicine & Biology*.

[10] Salvi P., Lio G., Labat C., Ricci E., Pannier B., Benetos A. (2004). Validation of a new non-invasive portable tonometer for determining arterial pressure wave and pulse wave velocity: the PulsePen device. *Journal of Hypertension*.

[11] Van Bortel L. M., Balkestein E. J., van der Heijden-Spek J. J. (2001). Non-invasive assessment of local arterial pulse pressure: comparison of applanation tonometry and echo-tracking. *Journal of Hypertension*.

[12] Avolio A. P., Van Bortel L. M., Boutouyrie P. (2009). Role of pulse pressure amplification in arterial hypertension: experts' opinion and review of the data. *Hypertension*.

[13] Boutouyrie P., Vermeersch S. J. (2010). Determinants of pulse wave velocity in healthy people and in the presence of cardiovascular risk factors: establishing normal and reference values. *European Heart Journal*.

[14] Lewis J. F., Kuo L. C., Nelson J. G., Limacher M. C., Quinones M. A. (1984). Pulsed Doppler echocardiographic determination of stroke volume and cardiac output: clinical validation of two new methods using the apical window. *Circulation*.

[15] Dinenno F. A., Tanaka H., Monahan K. D. (2001). Regular endurance exercise induces expansive arterial remodelling in the trained limbs of healthy men. *The Journal of Physiology*.

[16] Weber T., Ducos M., Mulder E. (2013). The specific role of gravitational accelerations for arterial adaptations. *Journal of Applied Physiology*.

[17] Convertino V. A., Doerr D. F., Mathes K. L., Stein S. L., Buchanan P. (1989). Changes in volume, muscle compartment, and compliance of the lower extremities in man following 30 days of exposure to simulated microgravity. *Aviation, Space, and Environmental Medicine*.

[18] Louisy F., Schroiff P., Güell A. (1997). Changes in leg vein filling and emptying characteristics and leg volumes during long-term head-down bed rest. *Journal of Applied Physiology*.

[19] Stout M. S., Watenpaugh D. E., Breit G. A., Hargens A. R. (1995). Simulated microgravity increases cutaneous blood flow in the head and leg of humans. *Aviation Space and Environmental Medicine*.

[20] Ferretti G., Iellamo F., Pizzinelli P. (2009). Prolonged head down bed rest-induced inactivity impairs tonic autonomic regulation while sparing oscillatory cardiovascular rhythms in healthy humans. *Journal of Hypertension*.

[21] Samijo S. K., Willigers J. M., Barkhuysen R. (1998). Wall shear stress in the human common carotid artery as function of age and gender. *Cardiovascular Research*.

[22] Weber T., Wassertheurer S., Hametner B. (2014). Reference values for central blood pressure. *Journal of the American College of Cardiology*.

